# Label-Free Imaging Analysis of Patient-Derived Cholangiocarcinoma Organoids after Sorafenib Treatment

**DOI:** 10.3390/cells11223613

**Published:** 2022-11-15

**Authors:** Michael Koch, Sandra Nickel, Ruby Lieshout, Susanna M. Lissek, Martina Leskova, Luc J. W. van der Laan, Monique M. A. Verstegen, Bruno Christ, Francesco Pampaloni

**Affiliations:** 1 Physical Biology, Buchmann Institute for Molecular Life Sciences (BMLS), Goethe University Frankfurt, 60438 Frankfurt am Main, Germany; 2 Department of Visceral, Transplant, Thoracic and Vascular Surgery, University of Leipzig Medical Center, 04103 Leipzig, Germany; 3 Division of General, Visceral and Vascular Surgery, University Hospital Jena, 07740 Jena, Germany; 4 Department of Surgery, Erasmus MC Transplant Institute, University Medical Center Rotterdam, 3015 CN Rotterdam, The Netherlands; 5 Experimental Medicine and Therapy Research, University of Regensburg, 93053 Regensburg, Germany

**Keywords:** label-free live imaging, brightfield microscopy, tumor organoids, primary liver cancer, intrahepatic cholangiocarcinoma, sorafenib

## Abstract

Monitoring tumor growth dynamics is crucial for understanding cancer. To establish an in vitro method for the continuous assessment of patient-specific tumor growth, tumor organoids were generated from patients with intrahepatic CCA (iCCA). Organoid growth was monitored for 48 h by label-free live brightfield imaging. Growth kinetics were calculated and validated by MTS assay as well as immunohistochemistry of Ki67 to determine proliferation rates. We exposed iCCA organoids (iCCAOs) and non-tumor intrahepatic cholangiocyte organoids (ICOs) to sub-therapeutic concentrations of sorafenib. Monitoring the expansion rate of iCCAOs and ICOs revealed that iCCAO growth was inhibited by sorafenib in a time- and dose-dependent fashion, while ICOs were unaffected. Quantification of the proliferation marker Ki67 confirmed inhibition of iCCAO growth by roughly 50% after 48 h of treatment with 4 µM sorafenib. We established a robust analysis pipeline combining brightfield microscopy and a straightforward image processing approach for the label-free growth monitoring of patient-derived iCCAOs. Combined with bioanalytical validation, this approach is suitable for a fast and efficient high-throughput drug screening in tumor organoids to develop patient-specific systemic treatment options.

## 1. Introduction

Bile duct cancers, or cholangiocarcinomas (CCAs), are rare but aggressive tumors arising from the biliary tree. CCAs have a very poor prognosis [1] and are categorized by anatomical location as intrahepatic (iCCA), perihilar (pCCA), or distal (dCCA). Liver resection or transplantation are the only curative treatment options for CCA [2]. However, the five-year survival is less than 10% [3] due to the high recurrence rate of 85% within the first three years after resection [4].

Today, non-resectable CCAs are treated with a combination of gemcitabine and cisplatin, extending median survival significantly by about 3.6 months [5]. Sorafenib, a multi-kinase inhibitor, has shown to be effective in the treatment of solid cancers such as renal cell carcinoma and hepatocellular carcinoma [6]. Although several studies showed a potential therapeutic beneficial effect, the therapeutic benefit in CCAs is still largely unknown [7,8,9].

Importantly, the interpatient heterogeneity of CCAs requires individual, patient-tailored systemic therapy to optimize the therapeutic outcome while simultaneously reducing adverse effects. However, appropriate procedures involving the patient-derived tumor tissue to screen for the most effective drug treatment are rarely available, if at all.

In addition to intrahepatic cholangiocyte organoids (ICOs) derived from the biliary compartment of the liver, an innovative CCA organoid model (CCAO) was recently established from resected tumor specimens and core tumor needle biopsies [10,11,12]. They were shown to preserve the majority of the mutational landscape and represent the epithelial cancer cell compartment of the original tumor to a large extent, leading to similar histopathology upon xenografting [10,11,13,14,15]. Moreover, they are suitable models for high-throughput drug screening approaches using luminescence or spectrophotometric endpoint readouts for cell viability [10,13,16].

Understanding biological systems such as (tumor) organoids and their dynamic behavior requires adequate techniques to investigate cellular dynamics under close-to-physiological conditions with minimal extrinsic perturbation [17,18]. To this aim, live imaging by advanced light microscopy together with subsequent advanced image analysis offer suitable approaches [19]. Different advanced light microscopy techniques are currently available that each hold unique advantages but also limitations with regards to in-depth resolution and invasiveness into the observed system [20].

To study organoid dynamics live at a global (entire culture) scale, brightfield microscopy presents a suitable solution for observing global changes in organoid size and morphology in multiple organoids simultaneously [21]. Brightfield microscopy offers a label-free, minimally invasive approach that can be easily adapted for high-throughput imaging. The reduced level of detail in the acquired image data allows for comparatively easy data handling and subsequent image analysis [22,23].

More detailed in-depth observations on the single-cell level can be achieved by fluorescence microscopy techniques such as confocal laser scanning microscopy (CLSM) [24] or light sheet-based fluorescence microscopy (LSFM) [25,26]. The vast amounts of data produced with these techniques, however, require considerable resources for post-processing and data evaluation [27,28].

In this project, we combined brightfield microscopy and an organoid-specific brightfield image analysis pipeline [21] to monitor the growth of patient-derived iCCAO treated with sorafenib, a drug currently not used for therapeutic intervention in cholangiocarcinoma.

## 2. Materials and Methods

### 2.1. Patient-Derived Liver Tissue and Organoid Initiation

Organoids were generated from one iCCA tissue sample collected after resection of the tumor for curative treatment (iCCAOs), and one healthy donor liver tissue sample (ICO) collected at the Erasmus MC—University Medical Center Rotterdam. The use of these tissue samples was approved by the Medical Ethics Committee of the Erasmus MC—University Medical Center Rotterdam (MEC-2013-143 and MEC-2014-060). All patients consented to the use of materials for research purposes. Organoids from iCCA biopsies and from healthy liver tissue were cultured as described previously by Broutier et al. [10] and Huch et al. [29]. The iCCAOs used in this study were characterized by Broutier et al. (originally labelled CC-1), and tumorigenicity was confirmed by xenografting and whole-exome sequencing [10].

### 2.2. Cell Proliferation Assay

The relative proliferation of iCCAOs was determined by CellTiter 96^®^ Aqueous One Solution Cell Proliferation Assay (MTS) according to the supplier’s instructions (CellTiter 96^®^ AQueous One Solution Cell Proliferation Assay, Technical Bulletin, version: revised 12/12, TB254, Promega, Walldorf, Germany). Organoids were dissociated into single cells by incubation in trypsin-EDTA (0.25%, Thermo Fisher Scientific, Dreieich, Germany) at 37 °C for five to ten min and vigorous pipetting. In total, 5 × 10^3^ organoid cells per well were seeded into 96-well plates (5 µL Matrigel™, Corning, Wiesbaden, Germany + 100 µL human liver expansion medium [30]). Cells were grown for seven days to form organoids before treatment with sorafenib (Santa Cruz Biotechnology, Heidelberg, Germany) for 72 h. Before the addition of 20 µL of CellTiter 96^®^ AQueous One Solution Reagent, the expansion/treatment medium was exchanged with 100 µL of basal medium [30]. Samples were incubated for one hour at standard culture conditions (37 °C, 5% CO_2_, humidified). The assay medium was transferred into a white/clear bottom 96-well plate and absorbance was measured on the microplate reader Tecan^®^ Infinite^®^ M200 with the corresponding software i-control™ (Tecan, Crailsheim, Germany).

### 2.3. Organoid Growth Analysis Pipeline—Organoid Size Measurements on Time-Resolved 2D Brightfield Image Data

The organoid growth analysis pipeline described here widely corresponds to the brightfield pipeline published in Hof et al. 2021 [21] with minor changes in sample preparation, image acquisition, and data analysis. In brief, organoids (iCCAOs and ICOs) were seeded into 96-well plates (5 µL Matrigel™ + 100 µL human liver expansion medium [30]), and grown for 72 h prior to image acquisition. The medium was exchanged and supplemented with the resp. compounds, where indicated, before starting the image acquisition. Organoids were imaged for 48 h with a recording interval of 0.5 h (≙97 time points, counting 0 h as time point 1) under controlled standard culture conditions (37 °C, 5% CO_2_, humidified).

Brightfield time-lapse recordings of organoids were acquired on the microscope AxioObserver.Z1 equipped with objective lens Plan-Apochromat 5×/0.16, camera AxioCam MR R3, and software ZEN blue (version 3.2, Carl Zeiss, Jena, Germany). Organoid cultures were imaged with 10 z-planes (z-spacing: 65 µm), 2 × 2 tiles (overlap: 15%, scan mode: meander), a pixel size of 1.29 × 1.29 μm^2^, an image size of 52,379 × 23,432 pixels, a bit depth of 12, an exposure time of 15 milliseconds (light source intensity: 3.22 V), and a binning mode of 1 × 1.

The recorded time-lapse image stacks were exported, converted from .czi to generic .tif format with software ZEN blue, and pre-processed with Fiji (ImageJ version 1.51n https://imagej.nih.gov/ij/download.html (accessed on 28 September 2022); Java version 1.8.0_6, 64-bit [31]). The dimensionality of the raw data sets was reduced from 4 (2 × 2) tiles with 10 z-planes each to one stitched image with one z-plane per time frame using the plugin Grid/Collection stitching [32]. The resulting image stacks were segmented based on projected luminal areas of the organoids by using the Fiji plugin Morphological Segmentation (MorphoLibJ [33]). Segmented luminal areas were measured with the Fiji plugin Region Morphometry (MorphoLibJ [33]). The luminal areas of individual organoids were normalized to the average luminal area over the first 5 time points. Organoids that were not detected in all 97 time frames were manually excluded from the analysis.

### 2.4. Immunohistochemistry

Organoids were fixed in paraformaldehyde and embedded in histogel (Thermo Fisher Scientific, Dreieich, Germany) followed by embedding in paraffin. Sections of 1 µm thickness were deparaffinized in the descending alcohol series. Epitopes were demasked for 30 min in citrate buffer, pH 6.0, using a pressure cooker. Following cooling for 30 min in ice water, endogenous peroxidases were blocked for 30 min with 3% H_2_O_2_ at 4 °C in the refrigerator. Sections were washed in PBS for 5 min, immediately followed by a 90 min BSA blocking step (5% BSA + 0.5% Tween20) in a humid chamber at RT. After applying the Vector-Kit SP-2001 according to the manufacturer’s manual (Vector Laboratories, Newark, CA, USA), slices were incubated overnight in a humid chamber at 4 °C with the mouse anti-human-Ki67 primary antibody (1:100, Dako M7240, Agilent Technologies Germany GmbH & Co., KG, Waldbronn, Germany). Following 3 washes with PBS for 5 min each, slices were incubated with the biotinylated goat anti-mouse secondary antibody (1:200, Dianova, Hamburg, Germany) for 1 h at RT in a humid chamber. After three additional washes with PBS, incubation was continued for 30 min with the ABC reagent (Vector Kit PK-6100; Vector Laboratories, Newark, CA, USA), and slices were counterstained with nuclear fast red for 3 min, followed by washing with PBS and subsequent incubation with Histogreen (Linaris GmbH, Dossenheim, Germany) for 2 min in a humid chamber. After the ascending alcohol series, the sections were embedded in Entellan (Merck GmbH, Darmstadt, Germany). For image acquisition, the microscope AxioImager.Z1 (Carl Zeiss, Jena, Germany) was applied.

For the detection of SOX9, the protocol as described was essentially the same, except that the primary rabbit anti-SOX9 antibody (1:200, ab185966, Abcam, Cambridge, UK) was used in combination with the biotinylated donkey anti-rabbit antibody (1:200, Dianova, Hamburg, Germany) as the secondary antibody.

Antigen demasking for immunofluorescence was achieved by 30 min incubation in Tris-EDTA buffer (pH 9.0). Subsequently, unspecific binding was blocked in 5% goat serum/PBS (ccpro, Oberdorla, Germany) for 20 min followed by 60 min in 5% BSA/0.5% Tween20. For the co-detection of E-cadherin and ZO-1, the mouse anti-E-cadherin (1:200, 610182, BD Biosciences, Heidelberg, Germany) and the rabbit anti-ZO-1 (1:100, 44-2200, InVitrogen, Carlsbad, CA, USA) antibodies were offered overnight in 1% BSA in PBS at 4 °C in a humid chamber. Following stepwise incubation with the goat anti-mouse Cy3 antibody (1:200, 115-165-003, Dianova, Hamburg, Germany) for 1 h, two washings for 10 min each in PBS, incubation with the goat anti-rabbit AlexaFluor 488 antibody (1:200, A11008, Life Technologies, Ober-Olm, Germany) for 1 h at RT, three washings for 5 min each in PBS, and nuclear counterstaining with DAPI (1:1000, Carl Roth GmbH + Co., KG, Karlsruhe, Germany) for 5 min, slices were finally embedded in 50% glycerol solution and lacquer. Images were taken using the microscope AxioObserver.Z1 (Oberkochen, Germany).

EpCAM and MDR1 were detected using the primary rabbit anti-EpCAM (1:200, ab213500, Abcam, Cambridge, England) and the rabbit anti-MDR1 (1:250, ab170904, Abcam, Cambridge, England) antibodies, resp., overnight at 4 °C in combination with the secondary goat anti-rabbit AlexaFluor 488 antibody (1:200, A11008, Life Technologies, Ober-Olm, Germany).

### 2.5. RNA Sequencing Analysis

Total RNA was isolated using TRIzol™ Reagent according to the supplier’s instructions (TRIzol™ Reagent User Guide, version: 28 January 2020, Thermo Fisher Scientific, Dreieich, Germany). In brief, organoids (iCCAOs and ICOs) were grown in 24-well plates (8 × 5 µL Matrigel™ droplets + 500 µL human liver expansion medium (PMID: 27560176)) for at least 7 days. Organoid-containing wells were rinsed with warm PBS (37 °C). Organoid material from two 24-wells was resuspended and combined in 1.5 mL of TRIzol™ Reagent. Volumes of reagents in the following steps were adjusted accordingly (×1.5). Lysates were vortexed vigorously for 30 sec and passed through a 20-G needle ten times before proceeding with the TRIzol™ protocol. RNA pellets were air-dried for at least 20 min and resuspended in 20 µL of UltraPure™ DNase/RNase-Free Distilled Water (Thermo Fisher Scientific, Dreieich, Germany). Samples were incubated at 55 °C for 15 min.

cDNA was synthesized from 1 µg of total RNA isolate using the Maxima First Strand cDNA Synthesis Kit for RT-qPCR according to the supplier’s instructions (Maxima First Strand cDNA Synthesis Kit for RT-qPCR Product Information, version: 2012, Thermo Fisher Scientific, Dreieich, Germany).

Transcripts of interest were amplified using KRAS-specific primers (Hs KRAS isoform A/B, forward primer: 5′-GAG AGA GGC CTG CTG AAA ATG-3′, reverse primer: 5′-CCC CGG CTC TCG GTT ATA AG-3′, biomers.net, Ulm, Germany) and Phusion™ High-Fidelity DNA Polymerase according to the supplier’s instructions (annealing temp.: 56.0 °C, 3-step protocol, Phusion™ High-Fidelity DNA Polymerase, Product Information Sheet, version: 28 January 2020, Thermo Fisher Scientific, Dreieich, Germany). Reaction volumes were scaled up to 100 µL containing 1 µL of undiluted cDNA template. To verify amplicon sizes, 20 µL of the PCR product were subjected to standard agarose-gel electrophoresis (2% agarose/TAE, 100 V, 30 min, TAE buffer: 40 mM Tris-HCl (pH 7.6), 20 mM acetic acid, 1 mM EDTA in ultrapure H_2_O).

In all, 80 µL of PCR product were cleaned-up with NucleoSpin^®^ Gel and PCR Clean-up according to the supplier’s instructions (5.1 PCR clean-up, NucleoSpin^®^ Gel and PCR clean-up, user manual, version: February 2017/Rev. 4, Machery-Nagel, Dueren, Germany). The PCR product was eluted in 20 µL of Buffer NE. A-tailing of cleaned-up blunt-end PCR product was achieved by incubation with Taq DNA Polymerase (Thermo Fisher Scientific, Dreieich, Germany) at 72 °C for 20 min (A-tailing reaction mix/10 µL: 1 µL of 10× PCR Buffer/-Mg, 0.25 µL of 50 mM MgCl2, 0.25 µL of 10 mM dATP, 8.25 µL of template cDNA, 0.25 µL of Taq DNA Polymerase).

Next, 3 µL of the A-tailed PCR product was ligated into the pGEM^®^-T Easy Vector System I according to the supplier’s instructions for 10 µL reactions (pGEM^®^-T and pGEM^®^-T Easy Vector Systems, Technical Manual, version: revised 12/18, TM042, Promega, Walldorf, Germany) at 16 °C overnight.

The ligation product was transformed into Subcloning Efficiency™ DH5α™ Competent Cells according to the supplier’s instructions with slight modifications using the entire volume (10 µL) of ligation product (Subcloning Efficiency™ DH5α™ Competent Cells, Product Information Sheet, version: 17 January 2006, Thermo Fisher Scientific, Dreieich, Germany). In brief, cells were heat-shocked for 30 sec at 42 °C. Transformed cells were incubated in 950 µL of SOC medium (New England BioLabs, Frankfurt am Main, Germany) at 37 °C and 225 rpm for one h.

Transformed cells were LB/ampicillin/IPTG/X-Gal plates (100 µg/mL ampicillin in LB agar, coated with 100 μL of 100 mM IPTG, and 20 μL of 50 mg/mL X-Gal, Carl Roth, Karlsruhe, Germany; according to pGEM^®^-T and pGEM^®^-T Easy Vector Systems, Technical Manual, version: revised 12/18, TM042, Promega, Walldorf, Germany). Plates were incubated for 16-24 h. White colonies were selected and incubated in 5 mL of LB medium + 100 µg/mL ampicillin (Carl Roth, Karlsruhe, Germany) at 37 °C and 225 rpm overnight. Amplified plasmid DNA was extracted with NucleoSpin^®^ Plasmid according to the supplier’s instructions (5.1 Isolation of high-copy plasmid DNA from *E. coli*, NucleoSpin^®^ Plasmid, User manual, version: March 2019/Rev. 11, Machery-Nagel, Dueren, Germany). Plasmid DNA was eluted in 30 µL of Buffer AE.

Insert sizes in amplified plasmid DNA were determined by restriction digest at 37 °C for one h (Restriction digest mix: 12.5 µL of nuclease-free H_2_O, 2 µL of rCutSmart™ Buffer, 5 µL of plasmid DNA, 0.5 µL of NotI-HF^®^, New England BioLabs, Frankfurt am Main, Germany). The digestion mix was subjected to standard agarose-gel electrophoresis to verify samples (2% agarose/TAE, 100 V, 30 min, TAE buffer: 40 mM Tris-HCl (pH 7.6), 20 mM acetic acid, 1 mM EDTA in ultrapure H_2_O).

Verified samples were diluted to 100 ng/µL in 20 µL final sample volume (Buffer AE, Machery-Nagel, Dueren, Germany) and sent for sequencing by Eurofins Genomics (SupremeRun Barcodes, GATC Services, Ebersberg, Germany). Sequencing data were analyzed with Geneious Prime^®^ (version 2021.1.1, Biomatters, Auckland, New Zealand).

### 2.6. Data Display and Statistical Analysis

Image data were processed with Fiji (ImageJ version 1.51n; Java version 1.8.0_6, 64-bit [31]) and displayed with PowerPoint^®^ (Microsoft; München, Germany).

Numerical data were cured with Excel^®^ (Microsoft, München, Germany), visualized with OriginPro^®^ 2020 (64-bit, SR1 9.7.0.188, Academic, OriginLab Corp., Northampton, MA, USA), and displayed with PowerPoint^®^. Normality or skewness in data distribution (descriptive statistics) was determined by Shapiro–Wilk testing in OriginPro^®^ 2020. Statistical analyses (hypothesis testing) were performed in OriginPro^®^ 2020. Data sets with small sample size and normal distribution were subjected to two-sample *t*-testing. Data sets with small sample size and skewed distribution were subjected to Mann–Whitney testing. Further details are given in the respective results sections.

Sequencing data were analyzed and visualized with Geneious Prime^®^ (version 2021.1.1, Biomatters, Auckland, New Zealand).

Statistics for the quantitative analysis of proliferation measured by Ki67 immunohistochemistry were performed using SPSS. For the non-normally distributed data set (Shapiro–Wilk), a Kruskal–Wallis test and post-hoc Bonferroni analysis of significance were performed. Values were considered as significantly different at the *P*-level as indicated.

## 3. Results

### 3.1. Cell Proliferation Assay Shows Dose-Dependent Susceptibility of iCCAOs to Multi-Kinase Inhibitor Sorafenib

A first screen with 133 compounds from an FDA-approved screening library showed intermediate to strong inhibitory effects of the multi-kinase inhibitor sorafenib on the established iCCAO line (results not shown). Additionally, previous studies also proposed a potentially beneficial effect of sorafenib in CCAs [6,7,8,9]. We therefore performed an exemplary MTS proliferation assay on the iCCAO line. We aimed to determine two concentrations of sorafenib for studying treatment effects at the morphological scale. This required efficacy without inducing high levels of apoptosis, which would impede long-term observations of morphological alterations under treatment conditions.

**Figure 1:**
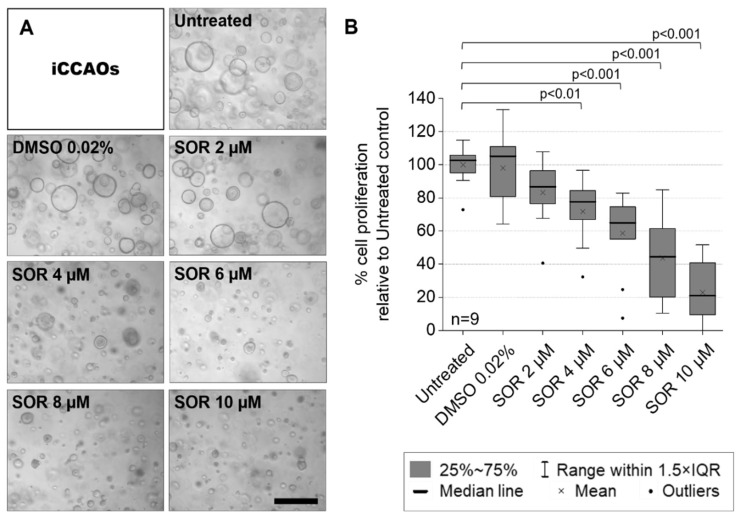
iCCAOs show dose-dependent decreases in cell proliferation under sorafenib treatment. Organoids were grown from single cells for seven days before treatment with sorafenib for 72 h. The vehicle control (DMSO 0.02%) corresponds to the vehicle concentration in 4 µM sorafenib. Vehicle controls for other conditions were excluded from display. (**A**) Phase-contrast images of organoids after treatment. Microscope: AxioVert 40 CFL; objective lens: EC-Achro-Plan 10×/0.5; camera: AxioCam ICm 1; pixel size: 1.29 × 1.29 μm^2^; scale bar: 1000 µm. (**B**) Cell proliferation was quantified by MTS assay [proliferation ≙ absorbance]. Proliferation values were normalized to the mean viability value of the untreated control. Statistics were performed on mean proliferation values [n = 9, normal distribution, two-sample *t*-test].

We determined 4 µM sorafenib to be the lowest effective concentration which significantly inhibits organoid growth over a treatment period of 72 h. We saw a drastic change in the morphological appearance of organoids (Figure 1A) and reduced mean proliferation by about 30% (Figure 1B). Accordingly, we decided to use 4 µM for brightfield microscopy experiments to further monitor and investigate changes in organoid growth and morphological behavior under treatment conditions. Although 2 µM sorafenib did not significantly decrease the MTS proliferation assay read-out, we still observed a reduction in mean proliferation of about 20% (Figure 1B). Therefore, we decided to use this concentration to investigate whether minor changes in morphological or growth behavior were elicited under prolonged treatment at half the lowest significantly effective concentration of 4 µM.

### 3.2. Organoid Growth Analysis Demonstrates Reduced Growth of iCCAOs upon Sorafenib Treatment

To study the effects of sorafenib treatment on organoid growth over time at a global morphological level by label-free, minimally invasive, and high-throughput means, we applied our recently published brightfield microscopy and corresponding image analysis pipeline [21]: here referred to as the organoid growth analysis (pipeline). We quantified organoid sizes in time-resolved brightfield data (48 h, recording interval: 0.5 h) of entire organoid cultures (on average 41–139 organoids per 96-well/5 µL Matrigel™) treated with 2 µM and 4 µM sorafenib.

**Figure 2:**
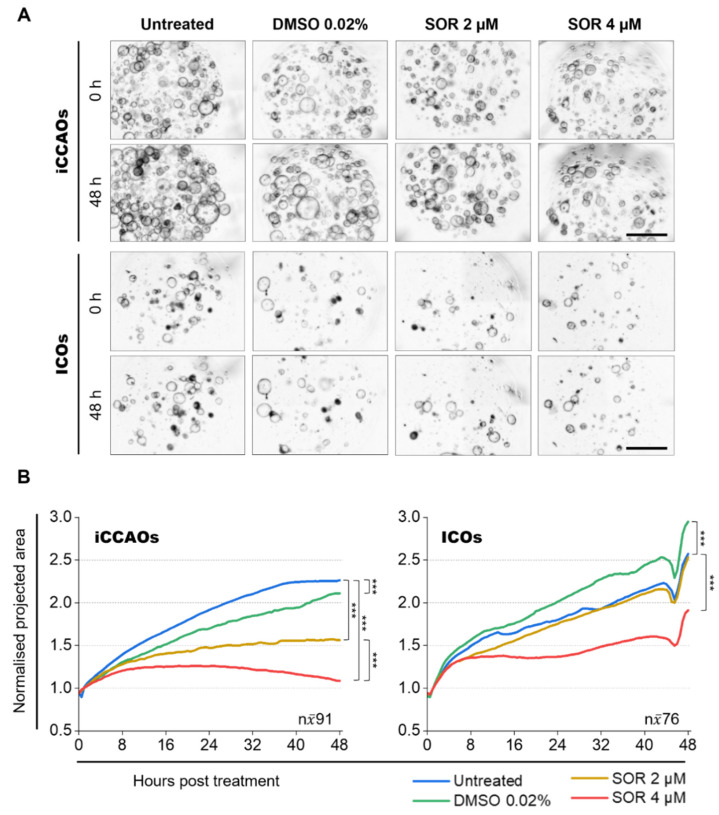
Sorafenib treatment limits iCCAO growth more extensively than ICO growth. (**A**) Overview of pre-processed (z-projected, stitched) brightfield images for subsequent segmentation and post-processing in the organoid growth analysis pipeline. Organoids were grown for 72 h prior to treatment. Post-treatment, organoid growth was monitored over 48 h with a recording interval of 0.5 h (≙ 97 time points, counting 0 h as time point 1). To capture the vast majority of organoids within one well (one Matrigel™ droplet), 2 × 2 tiles and 10 z-planes (z-spacing: 65 µm) were recorded with a magnification of 5×. Figure 2 shows representative stitched average intensity z-projections of n = 4 recordings for each organoid line and each condition. Microscope: AxioObserver.Z1; objective lens: Plan-Apochromat 5×/0.16; camera: AxioCam MR R3; pixel size: 1.29 × 1.29 μm^2^; scale bars: 1000 µm. (**B**) Quantification of organoid growth under sorafenib treatment. Data were generated with the organoid growth analysis pipeline. Graphs show median lines of normalized projected area (NPA) values for 97 consecutively measured time points (equivalent to recorded/analyzed time points: 48 h, 0.5 h recording interval ≙ 97 time points, counting 0 h as time point 1). Projected areas (PAs) for each organoid were normalized to their individual mean PA value over the first five time points. An overview and a comparison of relative final organoid size in NPA and percentage values are given in Table 1. For details on data distribution refer to Figures S1 and S2. Statistics were performed on median NPA values [n = 97, skewed distribution, Mann–Whitney test, *** *p* < 0.001].

By monitoring and quantifying the expansion of iCCAOs and ICOs under sorafenib treatment, we saw that growth in tumor organoids was generally more affected by sorafenib than growth in healthy organoids (Figure 2A,B). Compared to untreated organoids, relative sizes in tumor organoids after 48 h of treatment were significantly reduced to 71% at 2 µM sorafenib and 49% at 4 µM sorafenib. The vehicle control (DMSO 0.02%) showed a minor effect on tumor organoid growth with a size difference of 7%. When directly comparing the tumor organoid sizes at 2 µM and 4 µM sorafenib, we saw a dose-dependent size reduction of 31% (Figure 2B, Table 1).

ICOs showed a minor size reduction to 98% at 2 µM sorafenib but were highly affected by 4 µM sorafenib with a size reduction to 74% compared to untreated organoids. Surprisingly, the vehicle control (DMSO 0.02%) for ICOs showed a size increase of 15%. A direct comparison of organoid sizes at 2 µM and 4 µM sorafenib in ICOs further revealed a dose-dependent size reduction of 24% (Figure 2B).

Taken together, our results indicate that sorafenib inhibits growth more extensively in tumor organoids than in ICOs at low concentrations.

### 3.3. Determination of Proliferation and Apoptosis in iCCAOs by Immunohistochemistry

**Figure 3:**
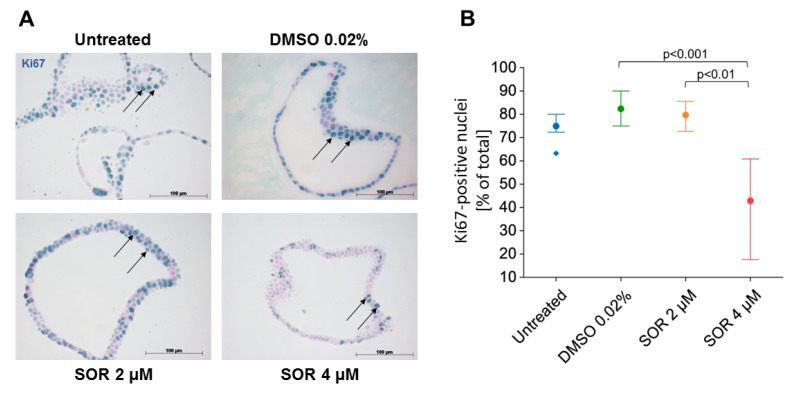
Sorafenib inhibits cell proliferation in iCCAOs in a concentration-dependent manner. (**A**) iCCAOs were treated for 48 h as indicated with the vehicle control (DMSO 0.02%), or with 2 and 4 µM sorafenib. Proliferating cells were identified by immunohistochemical detection of the nuclear antigen Ki67 (arrows). (**B**) For quantitative evaluation, positive cells in each 10 visual fields were detected using ImageJ and expressed as percentage number of total cells. Data were not normally distributed as verified by the Shapiro–Wilk test. The Kruskal–Wallis test and post-hoc Bonferroni analysis of significance were performed. Statistical analysis was accomplished using SPSS. Horizontal lines indicate the P-level of significance.

The determination of organoid growth inhibition by the organoid growth analysis pipeline as shown in Figure 2 was validated on the cellular level by immunohistochemical staining of the proliferation marker Ki67. As compared to untreated controls, the vehicle DMSO did not impact the number of Ki67-positive cells in the organoids, which was in the range of 80–90%. Treatment with 4 µM sorafenib for 48 h inhibited cell proliferation significantly by 50% (Figure 3A,B). This corroborates data determined by the organoid growth analysis pipeline, and moreover demonstrates that the growth of organoids is due to cell proliferation and not, e.g., to physical influences artificially “blowing up” the organoid size.

To show whether sorafenib inhibition of proliferation was associated with an increase in cell death, we stained iCCAOs for Caspase 3 activation. Yet, stained cells were found in the organoid tissue only occasionally. Stained cells were often visible in the organoid lumen, indicating extrusion of dying cells. Since these formed irregular conglomerates, quantification was not possible (Figure S3). Thus, whether cell death was due to normal turnover or due to sorafenib treatment cannot be decided unequivocally.

### 3.4. Expression of Functional Markers in iCCAOs after Sorafenib Treatment

**Figure 4:**
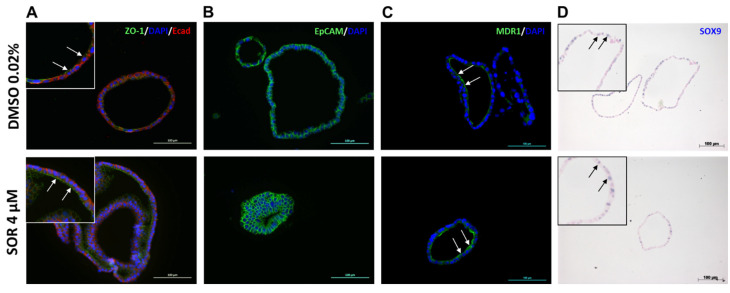
Sorafenib treatment does seemingly not affect protein expression of epithelial markers and proliferation marker SOX9 in iCCAOs. (**A**) iCCAOs were treated for 48 h as indicated either with the vehicle control (DMSO 0.02%), or with 4 µM sorafenib, resp. Fluorescent immunohistochemistry revealed apical and basolateral expression of ZO-1 (green) and E-cadherin (red), resp. Nuclei are labeled with DAPI (blue). Insets show digital magnifications (orig. magnification: 20×) of selected areas of the pictures behind. Arrows indicate luminal expression of ZO-1. (**B**) iCCAOs were treated for 48 h as indicated. Fluorescent immunohistochemistry showed membranous expression of EpCAM (green). Nuclei are labeled with DAPI (blue). (**C**) iCCAOs were treated for 48 h as indicated. Fluorescent immunohistochemistry revealed apical expression of MDR1 (green, white arrows). (**D**) iCCAOs were treated for 96 h as indicated. SOX9 expression was localized in the nuclei (insets, black arrows in digital magnifications (orig. magnification: 10×) of selected areas of the pictures behind) as shown by non-fluorescent immunohistochemistry.

The impact of sorafenib on functional features of iCCAOs was assessed by immunohistochemical detection of structural and functional markers after treatment with 4 µM sorafenib. The epithelial organization of the organoids was retained after treatment as indicated by the apical expression of the tight junction protein ZO-1 and the basolateral expression of the adherens junction protein E-cadherin (Figure 4A). This was supported by the expression of EpCAM, which was exclusively expressed in epithelial cells and played a role in tumorigenesis and metastasis [34], in both the control and sorafenib-treated organoids (Figure 4B). MDR1 is a bile export protein expressed in the liver at the apical membrane of hepatocytes and cholangiocytes [35]. This expression pattern was observed in the organoids after sorafenib treatment as demonstrated by the expression of MDR1 on the luminal side both in the controls and treated organoids (Figure 4C). The expression of SOX9, a transcription factor involved in proliferation, self-renewal, and tumorigenicity of cancer stem cells [36], was slightly, albeit not significantly, reduced by the treatment with sorafenib (Figure 4D and Figure S4). Taken together, the results suggest that protein expression patterns were largely similar in organoids with and without treatment with sorafenib.

### 3.5. RNA Sequencing Analysis Reveals Common KRAS Mutation in iCCAOs

To investigate whether the different sensitivities of ICOs and iCCAOs to sorafenib resulted from underlying mutational differences, we performed an RNA sequencing analysis of KRAS. This gene is commonly mutated in iCCA [37] and is involved in MAPK/ERK signaling, which is inhibited by sorafenib at the level of BRAF [38].

**Figure 5:**
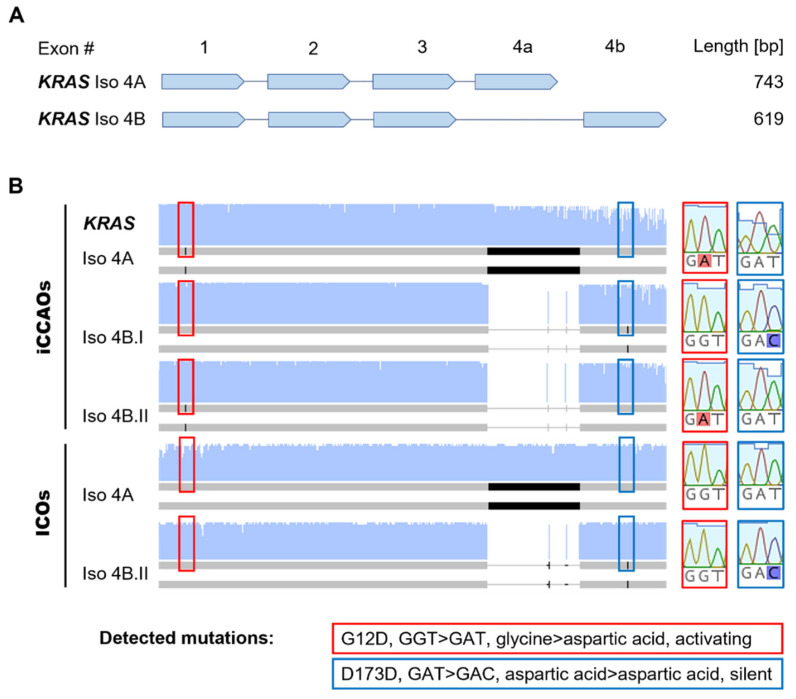
RNA sequencing analysis identifies activating G12D *KRAS* mutation in iCCAOs. (**A**) Simplified schematic overview of *KRAS* transcript variants with annotated lengths of corresponding PCR products. (**B**) Condensed overview of RNA sequencing analysis results for *KRAS* transcript variants in iCCAOs and ICOs. Red boxes indicate location of the detected activating G12D mutation and corresponding consensus sequence in non-mutated transcripts. Blue boxes indicate location of the detected silent D173D mutation and corresponding consensus sequence in non-mutated transcripts. Full sequences are listed in Table S1. Sequencing data were analyzed and visualized with Geneious Prime^®^ (version 2021.1.1, Biomatters).

iCCAOs expressed both isoforms of KRAS (Iso 4A, Iso 4B) described in the literature [39] (Figure 5A,B). Both isoforms exhibited the commonly occurring G12D mutation (GGT > GAT), in which glycine was replaced by aspartic acid constituting the activation of KRAS. This was also observed in the whole-exome sequencing of this organoid line performed earlier [10]. Additionally, we sequenced one transcript of Iso 4B (Iso 4B.I), which did not exhibit the G12D mutation but a rarely described silent D173D (GAT > GAC) mutation. These results indicate that both mutations are monoallelic.

ICOs also contained both isoforms of KRAS and expressed the wild-type form of Iso 4A (Figure 5). Only in one transcript of Iso 4B (Iso 4B.I), did we observe the aforementioned silent D173D mutation, indicating that this mutation is also monoallelic here.

Our results indicate that the mutational status of KRAS could partially explain why tumor organoids are more sensitive to sorafenib treatment than healthy organoids.

## 4. Discussion

### 4.1. Quantifying Organoid Growth in Patient-Derived Tumor Organoids by Label-Free Time-Lapse Brightfield Microscopy

We evaluated organoid growth under low-dose sorafenib treatment using our recently published brightfield microscopy-based image segmentation pipeline [21] (here referred to as: the organoid growth analysis pipeline). Our results show that this pipeline can be easily adapted to iCCAOs. This label-free imaging approach offers great potential for high-throughput screening of patient-derived organoid cultures [22,23,40,41]. However, detailed data from other disciplines such as proteomics [42] and transcriptomics [43] are needed to ascribe the morphologic changes observed over time to specific underlying cellular processes. For example, we need to know whether shrinking of the organoid is a mere mechanical phenomenon caused by deflation of the luminal volume, or if cell death is the driving underlying mechanism. Yet, the increase in organoid size can at least in part be attributed to proliferation as shown by the proliferation marker Ki67 (cf. Figure 3).

Regarding the technical aspects of the pipeline, the pre- and post-processing operations of the data still require a lot of manual work and need to be further optimized [21]. This will help to reduce the manual workload, and further push the pipeline towards a fully automated high-throughput image analysis approach. One tangible idea to improve the pre-processing steps is to adapt image acquisition, background subtraction, filtering, and binarizing to produce a better signal-to-noise ratio for subsequent image segmentation. In this project, we segmented and analyzed coherent, sequential time-resolved image stacks of organoid cultures observed for up to 48 h (recording interval: 0.5 h). However, we noticed that single time points are segmented more accurately than coherent time series (the smaller the series, the better the segmentation). Hence, another strategy to improve the segmentation process is to segment each time point separately and fuse the segmented time points afterwards for a subsequent analysis.

It is debatable whether the level of detail on organoid size change produced by the organoid growth analysis pipeline is required for evaluating treatment effects in patient-derived organoid cultures. To increase throughput and decrease the acquired amount of data, the temporal resolution can be decreased. However, we argue that even minor fluctuations in organoid size or morphology, which can be monitored especially under low-dose treatment conditions, enable detailed conclusions on individual treatment efficiency.

### 4.2. Understanding the Observed Morphological Treatment Effects in Patient-Derived Tumor Organoids

By quantifying organoid sizes in the time-resolved data of entire organoid cultures under low-dose sorafenib treatment, we observed that sorafenib had a higher impact on tumor organoid growth (for this particular patient) compared to growth in healthy organoids. To investigate whether these findings might result from underlying mutations in the MAPK pathway, which is targeted by sorafenib, we sequenced the KRAS gene [44]. KRAS is frequently mutated in iCCA [45]. While ICOs expressed the wild-type form of KRAS, we identified a common G12D mutation associated with constitutive activation of KRAS [46] and poor prognosis [37] in iCCAOs. Our results suggest that iCCA patients with this specific mutational background in KRAS might benefit from systemic low-dose sorafenib treatment as previously reported [47,48].

However, here, we only looked in detail at one specific component (KRAS) in a very complex system. Broader analyses by multi-omics approaches (e.g., next generation sequencing, mass spectrometry) [49,50] and integrating clinical patient characteristics are required to draw further conclusions on predictive parameters and paint a bigger picture on how the mutational background relates to morphological appearance/behavior and susceptibility to treatment [51,52].

## 5. Conclusions

The organoid growth analysis pipeline is a promising technique to measure drug response over time and could become a high-throughput predictive tool upon further validation both in vitro for more organoid lines and drugs, and by comparison to patient responses in vivo. The integration of results from multi-omics studies [51,52] could lead to more insight into the underlying substantiating network of predictive parameters to maximize interpretation of the acquired imaging data. Once this network of predictive parameters is established, high-throughput imaging approaches such as our label-free brightfield imaging pipeline can be used to obtain vast information on treatment effects without the need for further cross-validation by other disciplines.

## Figures and Tables

**Table 1:** Relative organoid sizes after 48 h of sorafenib treatment compared to untreated control. Percentages (right columns) were calculated from normalized projected area (NPA) values (left columns) obtained by organoid growth analysis (data shown in Figure 2). NPA values were rounded to two decimal places. Percentages were rounded to zero decimal places.

Condition	iCCAOs		ICOs	
	Determined NPA Value	NPA Relative to Control in %	Determined NPA Value	NPA Relative to Control in %
Untreated	2.27	100	2.57	100
DMSO 0.02%	2.10	93	2.95	115
SOR 2 µM	1.60	71	2.53	98
SOR 4 µM	1.11	49	1.91	74
SOR 4 µM/SOR 2 µM	---	69	---	76

## Data Availability

Data are contained within the article. Primary source data are available on request from the corresponding author.

## Supplementary Materials

The following supporting information can be downloaded at: https://www.mdpi.com/article/10.3390/cells11223613/s1, Figure S1: Data distribution of iCCAO growth upon sorafenib treatment; Figure S2: Data distribution of ICOs growth upon sorafenib treatment; Figure S3: Immunohistochemical detection of cleaved Caspase 3 in iCCAOs with and without sorafenib treatment; Figure S4: Image analysis of the immunohistochemical detection of SOX9 in iCCAOs with and without sorafenib treatment; Table S1: *KRAS* sequencing analysis nucleotide alignment.

## References

[1] DeOliveira M.L., Cunningham S.C., Cameron J.L., Kamangar F., Winter J.M., Lillemoe K.D., Choti M.A., Yeo C.J., Schulick R.D. (2007). Cholangiocarcinoma: Thirty-one-year experience with 564 patients at a single institution. Ann. Surg..

[2] Tautenhahn H.M., Bruckner S., Rauchfuss F., Donndorf F., Ardelt M., Fahrner R., Tannapfel A., Settmacher U. (2018). Precancerous and early stage cancer of the bile duct system. Chir. Z. Alle Geb. Oper. Medizen.

[3] Primrose J.N., Fox R., Palmer D.H., Prasad R., Mirza D., Anthoney D.A., Corrie P., Falk S., Wasan H.S., Ross P.J. (2017). Adjuvant capecitabine for biliary tract cancer: The bilcap randomized study. J. Clin. Oncol..

[4] Vogel A., Wege H., Caca K., Nashan B., Neumann U. (2014). The diagnosis and treatment of cholangiocarcinoma. Dtsch. Arztebl. Int..

[5] Valle J., Wasan H., Palmer D.H., Cunningham D., Anthoney A., Maraveyas A., Madhusudan S., Iveson T., Hughes S., Pereira S.P. (2010). Cisplatin plus gemcitabine versus gemcitabine for biliary tract cancer. N. Engl. J. Med..

[6] Wilhelm S.M., Carter C., Tang L., Wilkie D., McNabola A., Rong H., Chen C., Zhang X., Vincent P., McHugh M. (2004). Bay 43-9006 exhibits broad spectrum oral antitumor activity and targets the raf/mek/erk pathway and receptor tyrosine kinases involved in tumor progression and angiogenesis. Cancer Res..

[7] Luo X., Jia W., Huang Z., Li X., Xing B., Jiang X., Li J., Si A., Yang T., Gao C. (2017). Effectiveness and safety of sorafenib in the treatment of unresectable and advanced intrahepatic cholangiocarcinoma: A pilot study. Oncotarget.

[8] Pan T.T., Wang W., Jia W.D., Xu G.L. (2017). A single-center experience of sorafenib monotherapy in patients with advanced intrahepatic cholangiocarcinoma. Oncol. Lett..

[9] Chakunta H.R., Sunderkrishnan R., Kaplan M.A., Mostofi R. (2013). Cholangiocarcinoma: Treatment with sorafenib extended life expectancy to greater than four years. J. Gastrointest. Oncol..

[10] Broutier L., Mastrogiovanni G., Verstegen M.M., Francies H.E., Gavarro L.M., Bradshaw C.R., Allen G.E., Arnes-Benito R., Sidorova O., Gaspersz M.P. (2017). Human primary liver cancer-derived organoid cultures for disease modeling and drug screening. Nat. Med..

[11] Nuciforo S., Fofana I., Matter M.S., Blumer T., Calabrese D., Boldanova T., Piscuoglio S., Wieland S., Ringnalda F., Schwank G. (2018). Organoid models of human liver cancers derived from tumor needle biopsies. Cell Rep..

[12] Marsee A., Roos F.J.M., Verstegen M.M.A., Consortium H.P.B.O., Gehart H., de Koning E., Lemaigre F., Forbes S.J., Peng W.C., Huch M. (2021). Building consensus on definition and nomenclature of hepatic, pancreatic, and biliary organoids. Cell Stem Cell.

[13] Saito Y., Muramatsu T., Kanai Y., Ojima H., Sukeda A., Hiraoka N., Arai E., Sugiyama Y., Matsuzaki J., Uchida R. (2019). Establishment of patient-derived organoids and drug screening for biliary tract carcinoma. Cell Rep..

[14] Lampis A., Carotenuto P., Vlachogiannis G., Cascione L., Hedayat S., Burke R., Clarke P., Bosma E., Simbolo M., Scarpa A. (2018). Mir21 drives resistance to heat shock protein 90 inhibition in cholangiocarcinoma. Gastroenterology.

[15] Maier C.F., Zhu L., Nanduri L.K., Kuhn D., Kochall S., Thepkaysone M.L., William D., Grutzmann K., Klink B., Betge J. (2021). Patient-derived organoids of cholangiocarcinoma. Int. J. Mol. Sci..

[16] Li L., Knutsdottir H., Hui K., Weiss M.J., He J., Philosophe B., Cameron A.M., Wolfgang C.L., Pawlik T.M., Ghiaur G. (2019). Human primary liver cancer organoids reveal intratumor and interpatient drug response heterogeneity. JCI Insight.

[17] Lock J.G., Stromblad S. (2010). Systems microscopy: An emerging strategy for the life sciences. Exp. Cell Res..

[18] Mast F.D., Ratushny A.V., Aitchison J.D. (2014). Systems cell biology. J. Cell Biol..

[19] Rios A.C., Clevers H. (2018). Imaging organoids: A bright future ahead. Nat. Methods.

[20] Thorn K. (2016). A quick guide to light microscopy in cell biology. Mol. Biol. Cell.

[21] Hof L., Moreth T., Koch M., Liebisch T., Kurtz M., Tarnick J., Lissek S.M., Verstegen M.M.A., van der Laan L.J.W., Huch M. (2021). Long-term live imaging and multiscale analysis identify heterogeneity and core principles of epithelial organoid morphogenesis. BMC Biol..

[22] Buggenthin F., Marr C., Schwarzfischer M., Hoppe P.S., Hilsenbeck O., Schroeder T., Theis F.J. (2013). An automatic method for robust and fast cell detection in bright field images from high-throughput microscopy. BMC Bioinform..

[23] Kobayashi H., Lei C., Wu Y., Mao A., Jiang Y., Guo B., Ozeki Y., Goda K. (2017). Label-free detection of cellular drug responses by high-throughput bright-field imaging and machine learning. Sci. Rep..

[24] Elliott A.D. (2020). Confocal microscopy: Principles and modern practices. Curr. Protoc. Cytom..

[25] Pampaloni F., Chang B.J., Stelzer E.H. (2015). Light sheet-based fluorescence microscopy (lsfm) for the quantitative imaging of cells and tissues. Cell Tissue Res..

[26] Stelzer E.H.K., Strobl F., Chang B.-J., Preusser F., Preibisch S., McDole K., Fiolka R. (2021). Light sheet fluorescence microscopy. Nat. Rev. Methods Prim..

[27] Amat F., Hockendorf B., Wan Y., Lemon W.C., McDole K., Keller P.J. (2015). Efficient processing and analysis of large-scale light-sheet microscopy data. Nat. Protoc..

[28] Reynaud E.G., Peychl J., Huisken J., Tomancak P. (2015). Guide to light-sheet microscopy for adventurous biologists. Nat. Methods.

[29] Huch M., Gehart H., van Boxtel R., Hamer K., Blokzijl F., Verstegen M.M., Ellis E., van Wenum M., Fuchs S.A., de Ligt J. (2015). Long-term culture of genome-stable bipotent stem cells from adult human liver. Cell.

[30] Broutier L., Andersson-Rolf A., Hindley C.J., Boj S.F., Clevers H., Koo B.K., Huch M. (2016). Culture and establishment of self-renewing human and mouse adult liver and pancreas 3D organoids and their genetic manipulation. Nat. Protoc..

[31] Schindelin J., Arganda-Carreras I., Frise E., Kaynig V., Longair M., Pietzsch T., Preibisch S., Rueden C., Saalfeld S., Schmid B. (2012). Fiji: An open-source platform for biological-image analysis. Nat. Methods.

[32] Preibisch S., Saalfeld S., Tomancak P. (2009). Globally optimal stitching of tiled 3D microscopic image acquisitions. Bioinformatics.

[33] Legland D., Arganda-Carreras I., Andrey P. (2016). Morpholibj: Integrated library and plugins for mathematical morphology with imagej. Bioinformatics.

[34] Brown T.C., Sankpal N.V., Gillanders W.E. (2021). Functional implications of the dynamic regulation of epcam during epithelial-to-mesenchymal transition. Biomolecules.

[35] Jetter A., Kullak-Ublick G.A. (2020). Drugs and hepatic transporters: A review. Pharmacol. Res..

[36] Panda M., Tripathi S.K., Biswal B.K. (2021). Sox9: An emerging driving factor from cancer progression to drug resistance. Biochim. Biophys. Acta Rev. Cancer.

[37] Zhou S.L., Xin H.Y., Sun R.Q., Zhou Z.J., Hu Z.Q., Luo C.B., Wang P.C., Li J., Fan J., Zhou J. (2022). Association of kras variant subtypes with survival and recurrence in patients with surgically treated intrahepatic cholangiocarcinoma. JAMA Surg..

[38] Zhu Y.J., Zheng B., Wang H.Y., Chen L. (2017). New knowledge of the mechanisms of sorafenib resistance in liver cancer. Acta Pharmacol. Sin..

[39] Hobbs G.A., Der C.J., Rossman K.L. (2016). Ras isoforms and mutations in cancer at a glance. J. Cell Sci..

[40] Choo N., Ramm S., Luu J., Winter J.M., Selth L.A., Dwyer A.R., Frydenberg M., Grummet J., Sandhu S., Hickey T.E. (2021). High-throughput imaging assay for drug screening of 3d prostate cancer organoids. SLAS Discov. Adv. Life Sci. Drug Discov..

[41] Powell R.T., Moussalli M.J., Guo L., Bae G., Singh P., Stephan C., Shureiqi I., Davies P.J. (2022). Deeporganoid: A brightfield cell viability model for screening matrix-embedded organoids. SLAS Discov. Adv. Life Sci. Drug Discov..

[42] Krumm J., Sekine K., Samaras P., Brazovskaja A., Breunig M., Yasui R., Kleger A., Taniguchi H., Wilhelm M., Treutlein B. (2022). High temporal resolution proteome and phosphoproteome profiling of stem cell-derived hepatocyte development. Cell Rep..

[43] Brazovskaja A., Treutlein B., Camp J.G. (2019). High-throughput single-cell transcriptomics on organoids. Curr. Opin. Biotechnol..

[44] Liu P., Wang Y., Li X. (2019). Targeting the untargetable kras in cancer therapy. Acta Pharm. Sin. B.

[45] Haga H., Patel T. (2015). Molecular diagnosis of intrahepatic cholangiocarcinoma. J. Hepato Biliary Pancreat. Sci..

[46] Vatansever S., Erman B., Gumus Z.H. (2019). Oncogenic g12d mutation alters local conformations and dynamics of k-ras. Sci. Rep..

[47] LaRocca R.V., Hicks M.D., Mull L., Foreman B. (2007). Effective palliation of advanced cholangiocarcinoma with sorafenib: A two-patient case report. J. Gastrointest. Cancer.

[48] Pinter M., Sieghart W., Reisegger M., Wrba F., Peck-Radosavljevic M. (2011). Sorafenib in unresectable intrahepatic cholangiocellular carcinoma: A case report. Wien. Klin. Wochenschr..

[49] Hasin Y., Seldin M., Lusis A. (2017). Multi-omics approaches to disease. Genome Biol..

[50] Krassowski M., Das V., Sahu S.K., Misra B.B. (2020). State of the field in multi-omics research: From computational needs to data mining and sharing. Front. Genet..

[51] Codrich M., Dalla E., Mio C., Antoniali G., Malfatti M.C., Marzinotto S., Pierobon M., Baldelli E., Di Loreto C., Damante G. (2021). Integrated multi-omics analyses on patient-derived crc organoids highlight altered molecular pathways in colorectal cancer progression involving pten. J. Exp. Clin. Cancer Res..

[52] Lindeboom R.G., van Voorthuijsen L., Oost K.C., Rodriguez-Colman M.J., Luna-Velez M.V., Furlan C., Baraille F., Jansen P.W., Ribeiro A., Burgering B.M. (2018). Integrative multi-omics analysis of intestinal organoid differentiation. Mol. Syst. Biol..

