# Supplementary material for: Label-Free Imaging Analysis of Patient-Derived Cholangiocarcinoma Organoids after Sorafenib Treatment
Source: Cells. 2022 Nov 15;11(22):3613. doi: 10.3390/cells11223613 (PMC9688926; doi:10.3390/cells11223613)
Supplement: Supplementary file 1 [file cells-11-03613-s001.zip › Supplementary file 1_2022-11-01_clean.pdf]

Label-free imaging analysis of patient-derived cholangiocarcinoma organoids after sorafenib treatment

Michael Koch[1,†], Sandra Nickel[2,3,†], Ruby Lieshout[4], Susanna M. Lissek[5], Martina Leskova[1], Luc J.W. van der Laan[4], Monique M.A. Verstegen[4], Bruno Christ[2], and Francesco Pampaloni[1*]

Supplementary file 1

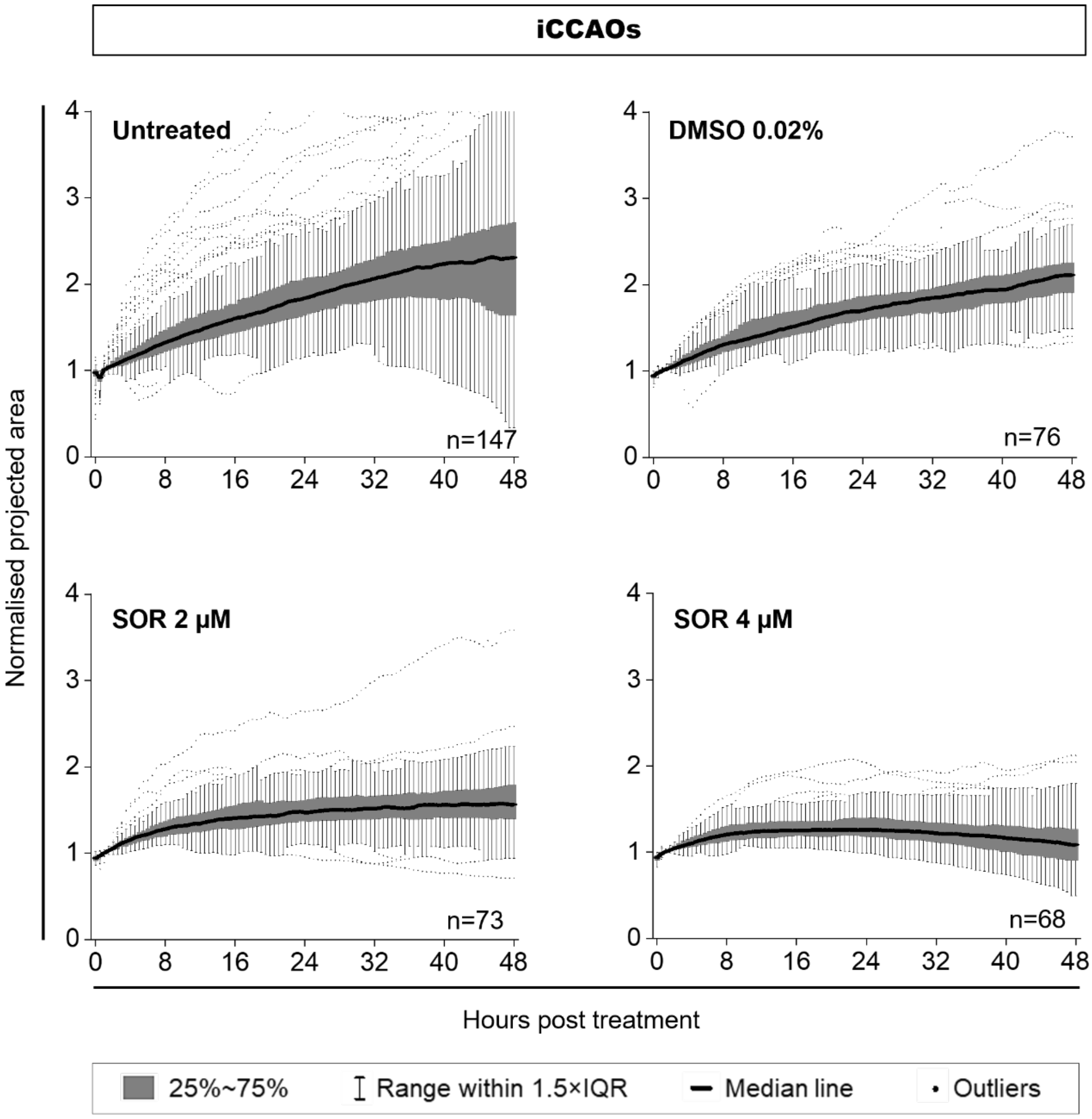

Figure S1. Data distribution of iCCAO growth upon sorafenib treatment. Details of data distribution for summarized median normalized projected area (NPA) values generated with the Organoid Growth Analysis pipeline, shown in Figure 2 in the main manuscript. Organoids were grown for 72 h prior to treatment. Projected areas (PAs) for each organoid were normalized to its individual mean PA value over the first five timepoints. Graphs show data for 97 consecutively measured timepoints (equivalent to recorded/analysed timepoints: 48 h, 0.5 h recording interval ≙ 97 timepoints, counting 0 h as timepoint 1). Outliers with NPA values larger than 4 were excluded from display for optimised visibility of the majority of data. Graphs were produced with OriginPro® 2020 (64-bit, SR1 9.7.0.188, Academic, OriginLab).

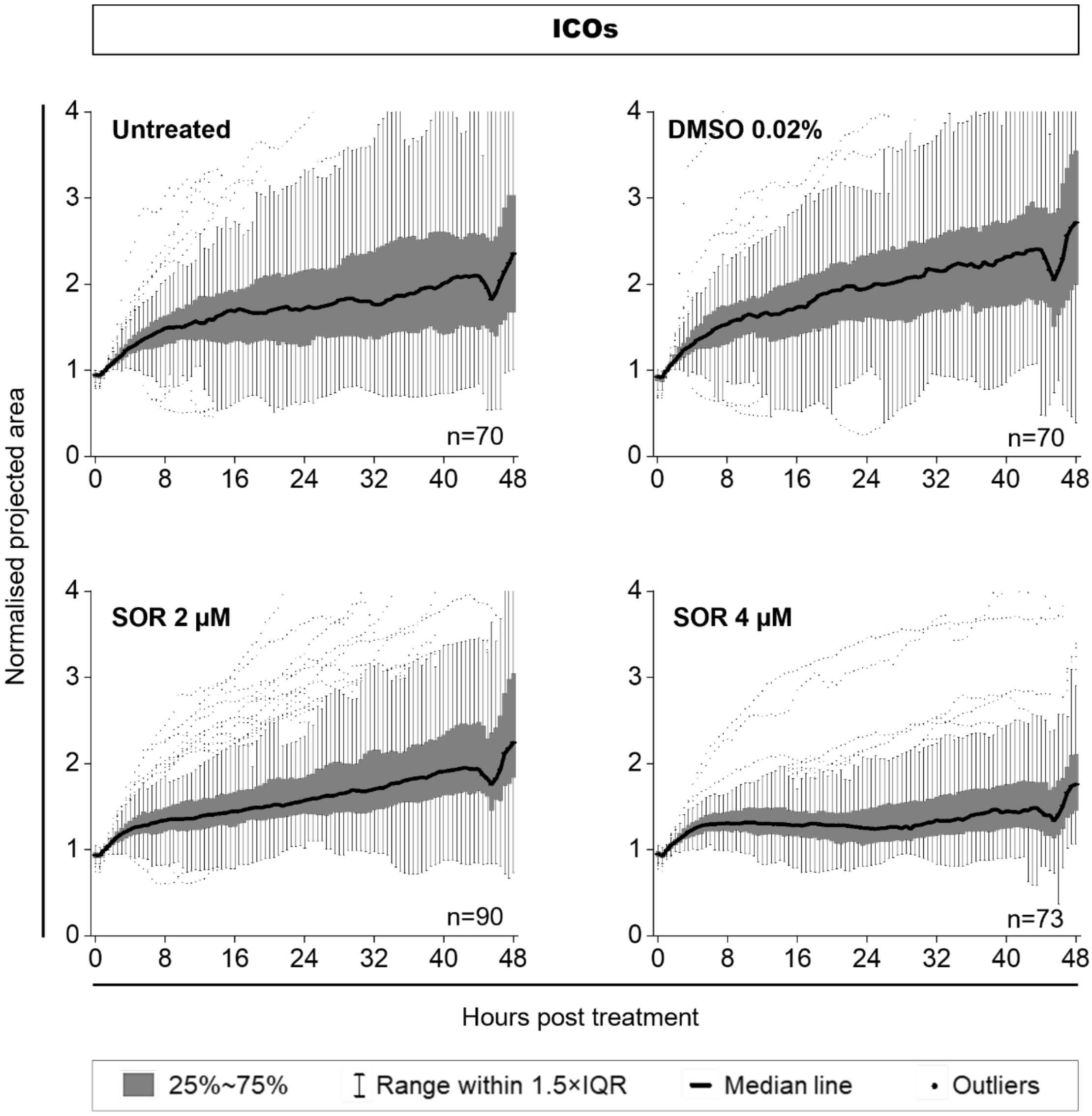

Figure S2. Data distribution of ICOs growth upon sorafenib treatment. Details of data distribution for summarized median normalized projected area (NPA) values generated with the Organoid Growth Analysis pipeline, shown in Figure 2 in the main manuscript. Organoids were grown for 72 h prior to treatment. Projected areas (PAs) for each organoid were normalized to its individual mean PA value over the first five timepoints. Graphs show data for 97 consecutively measured timepoints (equivalent to recorded/analysed timepoints: 48 h, 0.5 h recording interval ≙ 97 timepoints, counting 0 h as timepoint 1). Outliers with NPA values larger than 4 were excluded from display for optimised visibility of the majority of data. Graphs were produced with OriginPro® 2020 (64-bit, SR1 9.7.0.188, Academic, OriginLab).

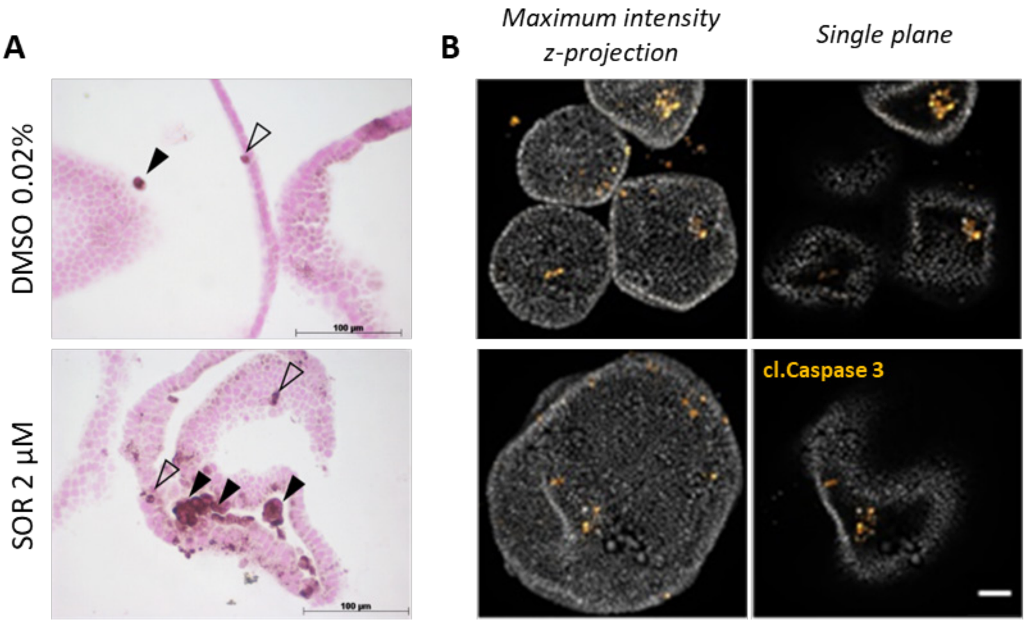

Figure S3. (Fluorescent) immunohistochemical detection of cleaved (cl.) Caspase 3 as an apoptosis marker in iCCAOs with and without sorafenib treatment. iCCAOs were incubated for 96 h in the presence of 2 µM sorafenib or 0.02% DMSO as the vehicle control. (**A**) Fixation and preparation of slices is described in the main manuscript. Cl.Caspase 3 was detected by using the rabbit anti-cl.Caspase 3 antibody (1:200; Cell Signaling Technology Europe, Leiden, The Netherlands) at 4 °C overnight. The biotinylated donkey anti-rabbit antibody (1:200, Dianova, Hamburg, Germany) was used as the secondary antibody and color developed with DAB Nova red and nuclear fast red as a counterstain. Note that only rare positive cells were detected in the organoid cell sheets (open arrowheads), while most dead cells were visible in the lumen as undefined conglomerates (solid arrowheads). (**B**) Organoids were prepared for whole-mount immunofluorescence staining as described by Broutier et al. [1]. Cl.Caspase 3 (active) was detected by using the rabbit anti-Caspase 3 (active) Asp175 antibody (1:400; Cell Signaling Technology Europe, Leiden, The Netherlands) at 4 °C overnight. The AlexaFluor™647-conjugated goat anti-rabbit antibody (1:400, Thermo Fisher Scientific, Dreieich, Germany) was used as the secondary antibody. Whole-mount staining confirmed findings that dead cells accumulated in the organoid lumen. Scale bar: 50 µm.

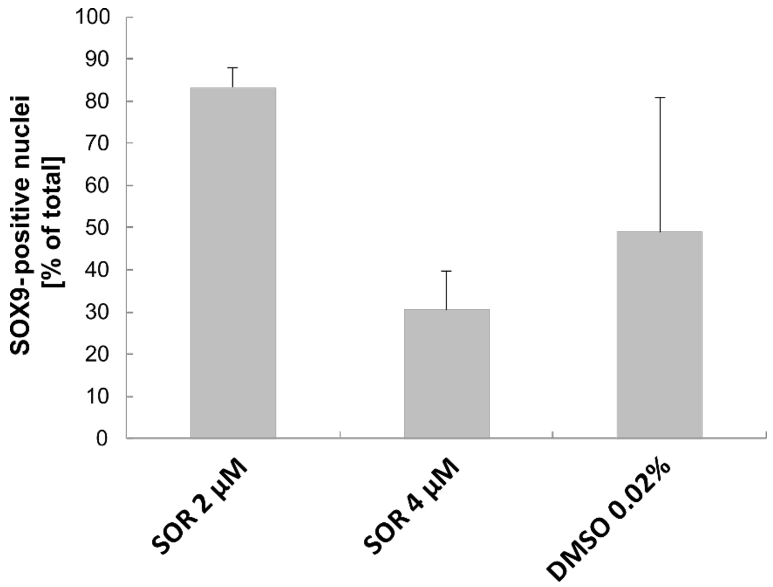

Figure S4. Image analysis of the immunohistochemical detection of SOX9 in iCCAOs with and without sorafenib treatment as shown representatively in Figure 4D in the main manuscript. iCCAOs were incubated for 96 h in the presence of 2 or 4 µM sorafenib, or 0.02% DMSO as the vehicle control. Organoids were fixed and slices prepared as described in the main manuscript. For the detection of SOX9, the primary rabbit anti-SOX9 antibody (1:200, ab185966, Abcam, Cambridge, England) was used in combination with the biotinylated donkey anti-rabbit antibody (1:200, Dianova, Hamburg, Germany) as the secondary antibody and Histogreen as the chromophore. Image acquisition was performed by using the Zeiss Axio Imager.Z1 microscope. For quantitative evaluation, positive nuclei in each 10 visual fields were detected using ImageJ and expressed as percentage number of total nuclei.

Table S1: *KRAS* sequencing analysis nucleotide alignment.

Sequence name	Sequence	Position
KRAS consensus seq.	**NGGAGAGSSGAGTCGCATGCTCCGGCCGCCATGGCGGCCGCGGGAATTCGATTGAGAGAG**	**60**
Iso 4A PCR Product	---GAGAGAG	7
Iso 4B PCR Product	---GAGAGAG	7
ICCAO Iso 4A	---AGAAGCGAGTCGCATGCTCCGGCCGCCATGGCGGCCGCGGGAATTCGATTGAGAGAG	57
ICCAO Iso 4B.I	-GGGTAGCGGAGTCGCATGCTCCGGCCGCCATGGCGGCCGCGGGAATTCGATTGAGAGAG	59
ICCAO Iso 4B.II	--GAAAGGCGAGTCGCATGCTCCGGCCGCCATGGCGGCCGCGGGAATTCGATTGAGAGAG	58
ICO Iso 4B.I	--NNNNNNNNNNTCGCANGCTCCGGCCGCCATGGCGGCCGCGGGAATTCGATTGAGAGAG	58
ICO Iso 4A	-NNNNNNNNNNNNCNCATGCTCCGGCCGCCATGGCGGCCGCGGGAATTCGATTGAGAGAG	59
ICO Iso 4B.II	NNNNNNNNNNNNNCNCATGCTCCGGCCGCCATGGCGGCCGCGGGA-TTCGATTGAGAGAG	59
KRAS consensus seq.	**GCCTGCTGAAAATGACTGAATATAAACTTGTGGTAGTTGGAGCTGGTGGCGTAGGCAAGA**	**120**
Iso 4A PCR Product	GCCTGCTGAAAATGACTGAATATAAACTTGTGGTAGTTGGAGCTGGTGGCGTAGGCAAGA	67
Iso 4B PCR Product	GCCTGCTGAAAATGACTGAATATAAACTTGTGGTAGTTGGAGCTGGTGGCGTAGGCAAGA	67
ICCAO Iso 4A	GCCTGCTGAAAATGACTGAATATAAACTTGTGGTAGTTGGAGCTGATGGCGTAGGCAAGA	117
ICCAO Iso 4B.I	GCCTGCTGAAAATGACTGAATATAAACTTGTGGTAGTTGGAGCTGGTGGCGTAGGCAAGA	119
ICCAO Iso 4B.II	GCCTGCTGAAAATGACTGAATATAAACTTGTGGTAGTTGGAGCTGATGGCGTAGGCAAGA	118
ICO Iso 4B.I	GCCTGCTGAAAATGACTGAATATAAACTTGTGGTAGTTGGAGCTGGTGGCGTAGGCAAGA	118
ICO Iso 4A	GCCTGCTGAAAATGACTGAATATAAACTTGTGGTAGTTGGAGCTGGTGGCGTAGGCAAGA	119
ICO Iso 4B.II	GCCTGCTGAAAATGACTGAATATAAACTTGTGGTAGTTGGAGCTGGTGGCGTAGGCAAGA	119
KRAS consensus seq.	**GTGCCTTGACGATACAGCTAATTCAGAATCATTTTGTGGACGAATATGATCCAACAATAG**	**180**
Iso 4A PCR Product	GTGCCTTGACGATACAGCTAATTCAGAATCATTTTGTGGACGAATATGATCCAACAATAG	127
Iso 4B PCR Product	GTGCCTTGACGATACAGCTAATTCAGAATCATTTTGTGGACGAATATGATCCAACAATAG	127
ICCAO Iso 4A	GTGCCTTGACGATACAGCTAATTCAGAATCATTTTGTGGACGAATATGATCCAACAATAG	177
ICCAO Iso 4B.I	GTGCCTTGACGATACAGCTAATTCAGAATCATTTTGTGGACGAATATGATCCAACAATAG	179
ICCAO Iso 4B.II	GTGCCTTGACGATACAGCTAATTCAGAATCATTTTGTGGACGAATATGATCCAACAATAG	178
ICO Iso 4B.I	GTGCCTTGACGATACAGCTAATTCAGAATCATTTTGTGGACGAATATGATCCAACAATAG	178
ICO Iso 4A	GTGCCTTGACGATACAGCTAATTCAGAATCATTTTGTGGACGAATATGATCCAACAATAG	179
ICO Iso 4B.II	GTGCCTTGACGATACAGCTAATTCAGAATCATTTTGTGGACGAATATGATCCAACAATAG	179
KRAS consensus seq.	**AGGATTCCTACAGGAAGCAAGTAGTAATTGATGGAGAAACCTGTCTCTTGGATATTCTCG**	**240**
Iso 4A PCR Product	AGGATTCCTACAGGAAGCAAGTAGTAATTGATGGAGAAACCTGTCTCTTGGATATTCTCG	187
Iso 4B PCR Product	AGGATTCCTACAGGAAGCAAGTAGTAATTGATGGAGAAACCTGTCTCTTGGATATTCTCG	187
ICCAO Iso 4A	AGGATTCCTACAGGAAGCAAGTAGTAATTGATGGAGAAACCTGTCTCTTGGATATTCTCG	237
ICCAO Iso 4B.I	AGGATTCCTACAGGAAGCAAGTAGTAATTGATGGAGAAACCTGTCTCTTGGATATTCTCG	239
ICCAO Iso 4B.II	AGGATTCCTACAGGAAGCAAGTAGTAATTGATGGAGAAACCTGTCTCTTGGATATTCTCG	238
ICO Iso 4B.I	AGGATTCCTACAGGAAGCAAGTAGTAATTGATGGAGAAACCTGTCTCTTGGATATTCTCG	238
ICO Iso 4A	AGGATTCCTACAGGAAGCAAGTAGTAATTGATGGAGAAACCTGTCTCTTGGATATTCTCG	239
ICO Iso 4B.II	AGGATTCCTACAGGAAGCAAGTAGTAATTGATGGAGAAACCTGTCTCTTGGATATTCTCG	239
KRAS consensus seq.	**ACACAGCAGGTCAAGAGGAGTACAGTGCAATGAGGGACCAGTACATGAGGACTGGGGAGG**	**300**
Iso 4A PCR Product	ACACAGCAGGTCAAGAGGAGTACAGTGCAATGAGGGACCAGTACATGAGGACTGGGGAGG	247
Iso 4B PCR Product	ACACAGCAGGTCAAGAGGAGTACAGTGCAATGAGGGACCAGTACATGAGGACTGGGGAGG	247
ICCAO Iso 4A	ACACAGCAGGTCAAGAGGAGTACAGTGCAATGAGGGACCAGTACATGAGGACTGGGGAGG	297
ICCAO Iso 4B.I	ACACAGCAGGTCAAGAGGAGTACAGTGCAATGAGGGACCAGTACATGAGGACTGGGGAGG	299
ICCAO Iso 4B.II	ACACAGCAGGTCAAGAGGAGTACAGTGCAATGAGGGACCAGTACATGAGGACTGGGGAGG	298
ICO Iso 4B.I	ACACAGCAGGTCAAGAGGAGTACAGTGCAATGAGGGACCAGTACATGAGGACTGGGGAGG	298
ICO Iso 4A	ACACAGCAGGTCAAGAGGAGTACAGTGCAATGAGGGACCAGTACATGAGGACTGGGGAGG	299

ICO Iso 4B.II	ACACAGCAGGTCAAGAGGAGTACAGTGCAATGAGGGACCAGTACATGAGGACTGGGGAGG	299
KRAS consensus seq.	**GCTTTCTTTGTGTATTTGCCATAAATAATACTAAATCATTTGAAGATATTCACCATTATA**	**360**
Iso 4A PCR Product	GCTTTCTTTGTGTATTTGCCATAAATAATACTAAATCATTTGAAGATATTCACCATTATA	307
Iso 4B PCR Product	GCTTTCTTTGTGTATTTGCCATAAATAATACTAAATCATTTGAAGATATTCACCATTATA	307
ICCAO Iso 4A	GCTTTCTTTGTGTATTTGCCATAAATAATACTAAATCATTTGAAGATATTCACCATTATA	357
ICCAO Iso 4B.I	GCTTTCTTTGTGTATTTGCCATAAATAATACTAAATCATTTGAAGATATTCACCATTATA	359
ICCAO Iso 4B.II	GCTTTCTTTGTGTATTTGCCATAAATAATACTAAATCATTTGAAGATATTCACCATTATA	358
ICO Iso 4B.I	GCTTTCTTTGTGTATTTGCCATAAATAATACTAAATCATTTGAAGATATTCACCATTATA	358
ICO Iso 4A	GCTTTCTTTGTGTATTTGCCATAAATAATACTAAATCATTTGAAGATATTCACCATTATA	359
ICO Iso 4B.II	GCTTTCTTTGTGTATTTGCCATAAATAATACTAAATCATTTGAAGATATTCACCATTATA	359
KRAS consensus seq.	**GAGAACAAATTAAAAGAGTTAAGGACTCTGAAGATGTACCTATGGTCCTAGTAGGAAATA**	**420**
Iso 4A PCR Product	GAGAACAAATTAAAAGAGTTAAGGACTCTGAAGATGTACCTATGGTCCTAGTAGGAAATA	367
Iso 4B PCR Product	GAGAACAAATTAAAAGAGTTAAGGACTCTGAAGATGTACCTATGGTCCTAGTAGGAAATA	367
ICCAO Iso 4A	GAGAACAAATTAAAAGAGTTAAGGACTCTGAAGATGTACCTATGGTCCTAGTAGGAAATA	417
ICCAO Iso 4B.I	GAGAACAAATTAAAAGAGTTAAGGACTCTGAAGATGTACCTATGGTCCTAGTAGGAAATA	419
ICCAO Iso 4B.II	GAGAACAAATTAAAAGAGTTAAGGACTCTGAAGATGTACCTATGGTCCTAGTAGGAAATA	418
ICO Iso 4B.I	GAGAACAAATTAAAAGAGTTAAGGACTCTGAAGATGTACCTATGGTCCTAGTAGGAAATA	418
ICO Iso 4A	GAGAACAAATTAAAAGAGTTAAGGACTCTGAAGATGTACCTATGGTCCTAGTAGGAAATA	419
ICO Iso 4B.II	GAGAACAAATTAAAAGAGTTAAGGACTCTGAAGATGTACCTATGGTCCTAGTAGGAAATA	419
KRAS consensus seq.	**AATGTGATTTGCCTTCTAGAACAGTAGACACAAAACAGGCTCAGGACTTAGCAAGAAGTT**	**480**
Iso 4A PCR Product	AATGTGATTTGCCTTCTAGAACAGTAGACACAAAACAGGCTCAGGACTTAGCAAGAAGTT	427
Iso 4B PCR Product	AATGTGATTTGCCTTCTAGAACAGTAGACACAAAACAGGCTCAGGACTTAGCAAGAAGTT	427
ICCAO Iso 4A	AATGTGATTTGCCTTCTAGAACAGTAGACACAAAACAGGCTCAGGACTTAGCAAGAAGTT	477
ICCAO Iso 4B.I	AATGTGATTTGCCTTCTAGAACAGTAGACACAAAACAGGCTCAGGACTTAGCAAGAAGTT	479
ICCAO Iso 4B.II	AATGTGATTTGCCTTCTAGAACAGTAGACACAAAACAGGCTCAGGACTTAGCAAGAAGTT	478
ICO Iso 4B.I	AATGTGATTTGCCTTCTAGAACAGTAGACACAAAACAGGCTCAGGACTTAGCAAGAAGTT	478
ICO Iso 4A	AATGTGATTTGCCTTCTAGAACAGTAGACACAAAACAGGCTCAGGACTTAGCAAGAAGTT	479
ICO Iso 4B.II	AATGTGATTTGCCTTCTAGAACAGTAGACACAAAACAGGCTCAGGACTTAGCAAGAAGTT	479
KRAS consensus seq.	**ATGGAATTCCTTTTATTGAAACATCAGCAAAGACAAGACAG-------------------**	**521**
Iso 4A PCR Product	ATGGAATTCCTTTTATTGAAACATCAGCAAAGACAAGACAGAGAGTGGAGGATGCTTTTT	487
Iso 4B PCR Product	ATGGAATTCCTTTTATTGAAACATCAGCAAAGACAAGACAG-------------------	468
ICCAO Iso 4A	ATGGAATTCCTTTTATTGAAACATCAGCAAAGACAAGACAGAGAGTGGAGGATGCTTTTT	537
ICCAO Iso 4B.I	ATGGAATTCCTTTTATTGAAACATCAGCAAAGACAAGACAG-------------------	520
ICCAO Iso 4B.II	ATGGAATTCCTTTTATTGAAACATCAGCAAAGACAAGACAG-------------------	519
ICO Iso 4B.I	ATGGAATTCCTTTTATTGAAACATCAGCAAAGACAAGACAG-------------------	519
ICO Iso 4A	ATGGAATTCCTTTTATTGAAACATCAGCAAAGACAAGACAGAGAGTGGAGGATGCTTTTT	539
ICO Iso 4B.II	ATGGAATTCCTTTTATTGAAACATCAGCAAAGACAAGACAG-------------------	520
KRAS consensus seq.	**--**	**521**
Iso 4A PCR Product	ATACATTGGTGAGGGAGATCCGACAATACAGATTGAAAAAAATCAGCAAAGAAGAAAAGA	547
Iso 4B PCR Product	--	468
ICCAO Iso 4A	ATACATTGGTGAGAGAGATCCGACAATACAGATTGAAAAAAATCAGCAAAGAAGAAAAGA	597
ICCAO Iso 4B.I	--	520
ICCAO Iso 4B.II	--	519
ICO Iso 4B.I	--	519
ICO Iso 4A	ATACATTGGTGAGAGAGATCCGACAATACAGATTGAAAAAAATCAGCAAAGAAGAAAAGA	599
ICO Iso 4B.II	--	520

KRAS consensus seq.	**---G-----------------------G-------------------TGTTGATGATGCC**	**536**
Iso 4A PCR Product	CTCCTGGCTGTGTGAAAATTAAAAAATGCATTATAATGTAATCTGGGTGTTGATGATGCC	607
Iso 4B PCR Product	---G-----------------------G-------------------TGTTGATGATGCC	483
ICCAO Iso 4A	CTCCTGGCTGTGTGAAAATTAAAAAATGCATTATAATGTAATCTGGGTGTTGATGATGCC	657
ICCAO Iso 4B.I	---G-----------------------G-------------------TGTTGATGATGCC	535
ICCAO Iso 4B.II	---G-----------------------G-------------------TGTTGATGATGCC	534
ICO Iso 4B.I	---G-----------------------G-------------------TGTTGATGATGCC	534
ICO Iso 4A	CTCCTGGCTGTGTGAAAATTAAAAAATGCATTATAATGTAATCTGGGTGTTGATGATGCC	659
ICO Iso 4B.II	-----G----------------------G------------------TGTTGATGATGCC	535
KRAS consensus seq.	**TTCTATACATTAGTTCGAGAAATTCGAAAACATAAAGAAAAGATGAGCAAAGATGGTAAA**	**596**
Iso 4A PCR Product	TTCTATACATTAGTTCGAGAAATTCGAAAACATAAAGAAAAGATGAGCAAAGATGGTAAA	667
Iso 4B PCR Product	TTCTATACATTAGTTCGAGAAATTCGAAAACATAAAGAAAAGATGAGCAAAGATGGTAAA	543
ICCAO Iso 4A	TTCTATACATTAGTTCGAGAAATTCGAAAACATAAAGAAAAGATGAGCAAAGATGGTAAA	717
ICCAO Iso 4B.I	TTCTATACATTAGTTCGAGAAATTCGAAAACATAAAGAAAAGATGAGCAAAGACGGTAAA	595
ICCAO Iso 4B.II	TTCTATACATTAGTTCGAGAAATTCGAAAACATAAAGAAAAGATGAGCAAAGATGGTAAA	594
ICO Iso 4B.I	TTCTATACATTAGTTCGAGAAATTCGAAAACATAAAGAAAAGATGAGCAAAGATGGTAAA	594
ICO Iso 4A	TTCTATACATTAGTTCGAGAAATTCGAAAACATAAAGAAAAGATGAGCAAAGATGGTAAA	719
ICO Iso 4B.II	TTCTATACATTAGTTCGAGAAATTCGAAAACATAAAGAAAAGATGAGCAAAGACGGTAAA	595
KRAS consensus seq.	**AAGAAGAAAAAGAAGTCAAAGACAAAGTGTGTAATTATGTAAATACAATTTGTACTTTTT**	**656**
Iso 4A PCR Product	AAGAAGAAAAAGAAGTCAAAGACAAAGTGTGTAATTATGTAAATACAATTTGTACTTTTT	727
Iso 4B PCR Product	AAGAAGAAAAAGAAGTCAAAGACAAAGTGTGTAATTATGTAAATACAATTTGTACTTTTT	603
ICCAO Iso 4A	AAGAAGAAAAAGAAGTCAAAGACAAAGTGTGTAATTATGTAAATACAATTTGTACTTTTT	777
ICCAO Iso 4B.I	AAGAAGAAAAAGAAGTCAAAGACAAAGTGTGTAATTATGTAAATACAATTTGTACTTTTT	655
ICCAO Iso 4B.II	AAGAAGAAAAAGAAGTCAAAGACAAAGTGTGTAATTATGTAAATACAATTTGTACTTTTT	654
ICO Iso 4B.I	AAGAAGAAAAAGAAGTCAAAGACAAAGTGTGTAATTATGTAAATACAATTTGTACTTTTT	654
ICO Iso 4A	AAGAAGAAAAAGAAGTCAAAGACAAAGTGTGTAATTATGTAAATACAATTTGTACTTTTT	779
ICO Iso 4B.II	AAGAAGAAAAAGAAGTCAAAGACAAAGTGTGTAATTATGTAAATACAATTTGTACTTTTT	655
KRAS consensus seq.	**TCTTAAGGCATACTAGAATCACTAGTGAATTCGCGGCCGCCTGCAGGTCGACCATATGGG**	**716**
Iso 4A PCR Product	TCTTAAGGCATACTAG--	787
Iso 4B PCR Product	TCTTAAGGCATACTAG--	663
ICCAO Iso 4A	TCTTAAGGCATACTAGAATCACTAGTGAATTCGCGGCCGCCTGCAGGTCGACCATATGGG	837
ICCAO Iso 4B.I	TCTTAAGGCATACTAGAATCACTAGTGAATTCGCGGCCGCCTGCAGGTCGACCATATGGG	715
ICCAO Iso 4B.II	TCTTAAGGCATACTAGAATCACTAGTGAATTCGCGGCCGCCTGCAGGTCGACCATATGGG	714
ICO Iso 4B.I	TCTTAAGGCATACTAGAATCACTAGTGAATTCGCGGCCGCCTGCAGGTCGACCATATGGG	714
ICO Iso 4A	TCTTAAGGCATACTAGAATCACTAGTGAATTCGCGGCCGCCTGCAGGTCGACCATATGGG	839
ICO Iso 4B.II	TCTTAAGGCATACTAGAATCACTAGTGAATTCGCGGCCGCCTGCAGGTCGACCATATGGG	715
KRAS consensus seq.	**AGAGCTCCCAACGCGTTGGATGCATAGCTTGAGTATTCTATAGTGTCACCTAAATAGCTT**	**776**
Iso 4A PCR Product	--	847
Iso 4B PCR Product	--	723
ICCAO Iso 4A	AGAGCTCCCAACGCGTTGGATGCATAGCTTGAGTATTCTATAGTGTCACCTAAATAGCTT	897
ICCAO Iso 4B.I	AGAGCTCCCAACGCGTTGGATGCATAGCTTGAGTATTCTATAGTGTCACCTAAATAGCTT	775
ICCAO Iso 4B.II	AGAGCTCCCAACGCGTTGGATGCATAGCTTGAGTATTCTATAGTGTCACCTAAATAGCTT	774
ICO Iso 4B.I	AGAGCTCCCAACGCGTTGGATGCATAGCTTGAGTATTCTATAGTGTCACCTAAATAGCTT	774
ICO Iso 4A	AGAGCTCCCAACGCGTTGGATGCATAGCTTGAGTATTCTATAGTGTCACCTAAATAGCTT	899
ICO Iso 4B.II	AGAGCTCCCAACGCGTTGGATGCATAGCTTGAGTATTCTATAGTGTCACCTAAATAGCTT	775

KRAS consensus seq.	**GGCGTAATCATGGTCATAGCTGTTTCCTGTGTGAAATTGTTATCCGCTCACAATTCCA-C**	**835**
Iso 4A PCR Product	--	907
Iso 4B PCR Product	--	783
ICCAO Iso 4A	GGCGTAATCATGGTCATAGCTGTTTCCTGTGTGAAATTGTTATCCGCTCACAATTCCACC	957
ICCAO Iso 4B.I	GGCGTAATCATGGTCATAGCTGTTTCCTGTGTGAAATTGTTATCCGCTCACAATTCCA-C	834
ICCAO Iso 4B.II	GGCGTAATCATGGTCATAGCTGTTTCCTGTGTGAAATTGTTATCCGCTCACAATTCCA-C	833
ICO Iso 4B.I	GGCGTAATCATGGTCATAGCTGTTTCCTGTGTGAAATTGTTATCCGCTCACAATTCCA-C	833
ICO Iso 4A	GGCGTAATCATGGTCATAGCTGTTTCCTGTGTGAAATTGTTATCCGCTCACAATTCCA-C	958
ICO Iso 4B.II	GGCGTAATCATGGTCATAGCTGTTTCCTGTGTGAAATTGTTATCCGCTCACAATTCCA-C	834
KRAS consensus seq.	**ACAACATACG-AGCCGGAAGCATAAAGTGTAAA--GCCTGGGGTGCCTAAT-GAGTGA-G**	**890**
Iso 4A PCR Product	--	967
Iso 4B PCR Product	--	843
ICCAO Iso 4A	ACAACATACG-AGCCGGAAGCATAAAGTGTAAA--GCCTGGGGTGCCTAAT-GAGTGA-G	1012
ICCAO Iso 4B.I	ACAACATACG-AGCCGGAAGCATAAAGTGTAAA--GCCTGGGGTGCCTAAT-GAGTGA-G	889
ICCAO Iso 4B.II	ACAACATACG-AGCCGGAAGCATAAAGTGTAAA--GCCTGGGGTGCCTAAT-GAGTGA-G	888
ICO Iso 4B.I	ACAACATACG-AGCCGGAAGCATAAAGTGTAAA--GCCTGGGGTGCCTAAT-GAGTGA-G	888
ICO Iso 4A	ACAACATACGAACCCGAAAGCATAAAGTGTAAAAGCCCGGGGGTGCCTAATGGAGNNANG	1018
ICO Iso 4B.II	ACAACATACG-AGCCGGAAGCATAAAGTGTAAA--GCCTGGGGTGCCTAAT-GAGTGA-G	889
KRAS consensus seq.	**CTAACTCACA-TTAATT-GCGTTGC-GCTCACTG-CCCGCT-TTCCAGT--CGGGAAACC**	**943**
Iso 4A PCR Product	--	1027
Iso 4B PCR Product	--	903
ICCAO Iso 4A	CTAACTCAACATTAATTGGCGTTGC-GCTCACTG-CCCGCTTT--CAGT--CGGGAAACC	1066
ICCAO Iso 4B.I	CTAACTCACA-TTAATT-GCGTTGC-GCTCACTG-CCCGCT-TTCCAGT--CGGGAAACC	942
ICCAO Iso 4B.II	CTAACTCACA-TTAATT-GCGTTGC-GCTCACTG-CCCGCT-TTCCAGT--CGGGAAACC	941
ICO Iso 4B.I	CTAACTCACA-TTAATT-GCGTTGC-GCTCACTG-CCCGCT-TTCCAGT--CGGGAAAAC	941
ICO Iso 4A	CTAACTCNCATTTAATTGGCGTTGCGGCTCACTGGCCCGCTTTTCCAGTTCCGGGAAACC	1078
ICO Iso 4B.II	CTAACTCACA-TTAATT-GCGTTGC-GCTCACTG-CCCGCT-TTCCAGT--CGGGAAACC	942
KRAS consensus seq.	**T-GTCGT-GCCAGC-TGCAT-TAATG-AATC-GGCCAACGCGC-GGGG--AAGAGGCGG-**	**993**
Iso 4A PCR Product	--	1087
Iso 4B PCR Product	--	963
ICCAO Iso 4A	TTGTCGGTGCCAGC-TGCATTAAATG--ATC--GACAACGCGG-CGGGGAGAGAGGCGA-	1119
ICCAO Iso 4B.I	T-GTCGT-GCCAGC-TGCAT-TAATG-AATC-GGCCAACGCGC-GGGG---AGAGGCGG-	991
ICCAO Iso 4B.II	T-GTCGT-GCCAGC-TGCAT-TAATG--ATC-GGCCAACGCGC-GGGG---AGAGGCGGT	990
ICO Iso 4B.I	CTGTCGT-GCCAGC-TGCAT-TAATG-AATC-GGCCAACGCGC-GGGGG-AGAAGGCGGG	994
ICO Iso 4A	TGGTCGTGGCCAGCTTGCATTTATTGAAATCGGGCCAACGCCCNGGGGGAAAAAGGCGGT	1138
ICO Iso 4B.II	T-GTCGT-GCCAGC-TGCAT-TAATG-AATC-GGCCAACGCNNGGGG---AAGAGGCGG-	992
KRAS consensus seq.	**TTTGCGT-ATTGGGGCGC-TC-TTCCGCTT--CCTCGCTC-ACTGA--CTCGCT-GCGC-**	**1043**
Iso 4A PCR Product	--	1147
Iso 4B PCR Product	--	1023
ICCAO Iso 4A	TTTGCGT-AT--GGGCGC-TC-TCCGGCTT---CTCGCTCACTTGA--CTCGCTTGCGCT	1169
ICCAO Iso 4B.I	TTTGCGT-ATTGGGGCGCTTC-TTCCGCTTT--CTCGCTC-ACTGA--CTCGCT-GCGC-	1042
ICCAO Iso 4B.II	TTGCCGTA--TGGGGCGCTTC-T-CCGCTT--CCTCGCTC-ACTGA--CTCGCTGGCGC-	1040
ICO Iso 4B.I	TTGGCGT-ATTGGGGCGCTTC-TTCCGCTT-CCCTCGCTC-ACTGAA-CTCGCTTCGGC-	1048
ICO Iso 4A	TTTGCCTAATTGGGGCNCCTCTTTCCGCTTTCCCTCGNTTAAATGAAACTCGGTTG-GCG	1197
ICO Iso 4B.II	TTTGCGT-ATTGGG-CGC-TC-TTCCGCTT--CCTCGCTC-ANTGA--CTCGCT-GCGC-	1041
KRAS consensus seq.	**--TC-GGTCGTT-CGGCTGCGGCGAAGCGG-TATCAGCT-CACTCAAA-GGC-GGN-AA**	**1094**

Iso 4A PCR Product	--	1207
Iso 4B PCR Product	--	1083
ICCAO Iso 4A	CGTC-GGTCG-----GCTG-GCGCGAAGCGAT--TCAGCT-CACTCAA--GGC-G---TA	1213
ICCAO Iso 4B.I	--TC-GGTCGT---CGCTGCGG-CGA-GCGGTTATCAGCT-CACTCAAAGGGC-GT--AA	1090
ICCAO Iso 4B.II	--TC-GGTCGT---CGCTGCGG-CGA-GCGTAT-TCAGCT-CACTCGA--GGC-GGT--A	1085
ICO Iso 4B.I	--TCGGGTCGTTCGGGCTGCCGGCGAAGCGGGTATCAGCTTCACTCCAAAGGCGGGTAAA	1106
ICO Iso 4A	CTTCGGGTCGTTTCA---	1257
ICO Iso 4B.II	--TC-GGTCGTT-CGGCTGCGGGC-AAGCGG-TATCAGCT-CACTCAAAGGGCGGT--AA	1092
KRAS consensus seq.	**TACGG-TTATCC--ACAGAAATCAGGGG-A-WAACGCAGG-AAAAGAACAT-GTGA--CC**	**1145**
Iso 4A PCR Product	--	1267
Iso 4B PCR Product	--	1143
ICCAO Iso 4A	TCCGG--TATTC--TCAG-ATTCAGGG--A-TTACGCGAGTAAGAAACCGT-GTGA--CC	1262
ICCAO Iso 4B.I	TACGG--TATTCCCACAG-ATTCAGGG----ATACGCAG----AAGAACAT-GTGA--GC	1136
ICCAO Iso 4B.II	TACGGGTTATTC--ACAGAA-TCAGG---A-TTACGCAG----AAGAAACTTGTGA--GC	1132
ICO Iso 4B.I	TACGGGTTATCCCC-CAGAAATCCGGGGGAAAAACGCAGGGAAAAGAACATGGTGAAGCC	1165
ICO Iso 4A	--	1317
ICO Iso 4B.II	TACGGGTTATCC--ACAGAAATCAGGGGGATAAANCCAGG-AAAAAAACAT-GTGGAGCC	1148
KRAS consensus seq.	**AAAAGGGCCAGCMAAA--GGCCAGGAARACCGTN--AAAAGGCC-------GGGTGCTGG**	**1194**
Iso 4A PCR Product	--	1327
Iso 4B PCR Product	--	1203
ICCAO Iso 4A	AAAAGGGCTG--	1322
ICCAO Iso 4B.I	AAA--GGCCAGCTAAG-------GCAAGAACGT----AAAGGCC-------GGATGCTGC	1176
ICCAO Iso 4B.II	AA----GTCAGCAAA---------GCAGATCGTT--AAAAGCCC---------GTGCTGG	1168
ICO Iso 4B.I	AAAAGGGCCAGCCAAAAGGGCCAAGGAAACCGTTAAAAAAGGGCCCGCNTTTGGCTGGGG	1225
ICO Iso 4A	--	1377
ICO Iso 4B.II	AAAAGGCCCA--	1208
KRAS consensus seq.	**CGTTTTTCCATAGBCCCVCTCTGACAGACVAVTADHDCCAAAGTGACGGTTVGCDAAVVV**	**1254**
Iso 4A PCR Product	--	1387
Iso 4B PCR Product	--	1263
ICCAO Iso 4A	--	1382
ICCAO Iso 4B.I	GATTTTTCCATAGCTCCGCTCTGACAGAACTCTAATTGCAGCGTCTCAGGTCCCGAGAGG	1236
ICCAO Iso 4B.II	CCATTTCATAGCCTCCGACTCTGACACTCAAATCGAGCCTAAGTGAAGGTTGGCAAACCC	1228
ICO Iso 4B.I	CGTTTTTTCA--	1285
ICO Iso 4A	--	1437
ICO Iso 4B.II	--	1268
KRAS consensus seq.	**BGTVGCGAAACYCGWSSSACTMWRAGAATCAGGCGTTTCCCCTTGAGCTCCCTCGGTGGC**	**1314**
Iso 4A PCR Product	--	1447
Iso 4B PCR Product	--	1323
ICCAO Iso 4A	--	1442
ICCAO Iso 4B.I	TGTCGCGAAACTCGTGCCACTCAG------------------------------------	1296
ICCAO Iso 4B.II	CGTACG--	1288
ICO Iso 4B.I	--	1345
ICO Iso 4A	--	1497
ICO Iso 4B.II	--	1328

Reference

1. Broutier, L.; Andersson-Rolf, A.; Hindley, C.J.; Boj, S.F.; Clevers, H.; Koo, B.K.; Huch, M., Culture and establishment of self-renewing human and mouse adult liver and pancreas 3d organoids and their genetic manipulation. Nature protocols 2016, 11, 1724-1743.
